# DNA Barcoding of the Endangered *Aquilaria* (Thymelaeaceae) and Its Application in Species Authentication of Agarwood Products Traded in the Market

**DOI:** 10.1371/journal.pone.0154631

**Published:** 2016-04-29

**Authors:** Shiou Yih Lee, Wei Lun Ng, Mohd Noor Mahat, Mohd Nazre, Rozi Mohamed

**Affiliations:** 1 Forest Biotech Laboratory, Department of Forest Management, Universiti Putra Malaysia, 43400 UPM Serdang, Selangor, Malaysia; 2 Institute of Bioscience, Universiti Putra Malaysia, 43400 UPM Serdang, Selangor, Malaysia; 3 Forest Research Institute Malaysia, 52109 Kepong, Selangor, Malaysia; 4 Department of Forest Management, Universiti Putra Malaysia, 43400 UPM Serdang, Selangor, Malaysia; Chinese Academy of Medical Sciences, Peking Union Medical College, CHINA

## Abstract

The identification of *Aquilaria* species from their resinous non-wood product, the agarwood, is challenging as conventional techniques alone are unable to ascertain the species origin. *Aquilaria* is a highly protected species due to the excessive exploitation of its precious agarwood. Here, we applied the DNA barcoding technique to generate barcode sequences for *Aquilaria* species and later applied the barcodes to identify the source species of agarwood found in the market. We developed a reference DNA barcode library using eight candidate barcode loci (*mat*K, *rbc*L, *rpo*B, *rpo*C1, *psb*A-*trn*H, *trn*L-*trn*F, ITS, and ITS2) amplified from 24 leaf accessions of seven *Aquilaria* species obtained from living trees. Our results indicated that all single barcodes can be easily amplified and sequenced with the selected primers. The combination of *trn*L-*trn*F+ITS and *trn*L-*trn*F+ITS2 yielded the greatest species resolution using the least number of loci combination, while *mat*K+*trn*L-*trn*F+ITS showed potential in detecting the geographical origins of *Aquilaria* species. We propose *trn*L-*trn*F+ITS2 as the best candidate barcode for *Aquilaria* as ITS2 has a shorter sequence length compared to ITS, which eases PCR amplification especially when using degraded DNA samples such as those extracted from processed agarwood products. A blind test conducted on eight agarwood samples in different forms using the proposed barcode combination proved successful in their identification up to the species level. Such potential of DNA barcoding in identifying the source species of agarwood will contribute to the international timber trade control, by providing an effective method for species identification and product authentication.

## Introduction

*Aquilaria* Lam., an endangered genus in the family Thymelaeaceae, is well known for its production of a fragrant non-wood product generally known as ‘agarwood’. Demand for agarwood is high in the international market due to its scarcity, which escalates its market price. To produce agarwood, destructive processes such as mechanical hacking, slashing, cork boring, to name a few, are inflicted onto the tree’s woody part to create wounds for fungal infection to set in, thus inducing the formation of agarwood [1,2]. While not all *Aquilaria* species can produce agarwood effectively through mechanical wounding, indiscriminate harvesting of agarwood from the wild had threatened the survival of these trees. Over-exploitation of these trees in the wild has resulted in nine *Aquilaria* species being listed in the Red List of Threatened Species in the year 2010 by the International Union for Conservation of Nature (IUCN). The genus *Aquilaria* was further classified in Appendix II of the Convention on International Trade in Endangered Species of Fauna and Flora (CITES) as “Endangered”. This effectively placed all species in the entire *Aquilaria* genus under CITES protection, which among others requires trade permits for export purposes [3]. These classifications demonstrate that the agarwood trade is closely monitored by international regulators to ensure that such activity will not continue to be detrimental to the continued existence of these species in the wild.

Agarwood is used in its original form for many traditional practices and as an essential raw material for the production of many consumer products. The use of agarwood started as early as 2,000 years ago; until today the demand for agarwood continues to grow. The possession of agarwood was once restricted to the inner circles within the imperial palaces and high-ranked officers, but it is now affordable to regular people. In China, agarwood is known as *chenxiang*, and is an important ingredient in traditional medicines and has been used to relieve spasms in human digestive and respiratory systems [4]. In Japan, it is known as *jinkoh* and is burnt in appreciation ceremonies and during meditation [5]. The Arabs apply essential oil from agarwood (*oud*) the same way as using perfumes or fragrance oil [6]. In other parts of the world, agarwood is a core material in religious applications such as in the making of incenses, religious carvings, and accessories. Nowadays, big chunks of agarwood are being sought-after by the rich, as it has become trendy to own agarwood artifacts of a variety of shapes and sizes.

At present, there are 21 recorded *Aquilaria* species and they are widely distributed in the Indo-Malesian region, spanning over 12 countries [7,8]. Of all species, only a handful are being exploited due to their wide occurrence in the wild, including *A*. *beccariana*, *A*. *crassna*, *A*. *filaria*, *A*. *hirta*, *A*. *malaccensis*, *A*. *microcarpa*, *A*. *sinensis*, and *A*. *subintegra* [9–13]. In the agarwood industry, consumer preference is often influenced by the geographical origin of the agarwood, which is generally indicative of the species growing in that region and hence the supposed ‘quality’ of an agarwood product. This also determines their market prices, as traders and potential buyers believe agarwood from different *Aquilaria* species bear distinctive fragrances and medicinal attributes. For example, *A*. *sinensis* is recognized as the best agarwood source for use in Chinese traditional medicines, and *A*. *malaccensis* as the only known imported source of acceptable properties for the same purpose [4]. In Japan, agarwood from *A*. *crassna* is preferred for appreciation ceremonies due to the particular sweetness when burnt [5]. The Arabs on the other hand, prefer essential oils extracted from *A*. *malaccensis* because of the strong fragrance compared to the other *Aquilaria* species [6]. *A*. *sinensis* and *A*. *crassna* are also preferred over the other species as sources of carving material due to personal affection towards the wood structure and its fragrance. Since identification of *Aquilaria* species is mainly based on floral and fruit characteristics of the tree, CITES has suggested for improvements in the identification methods [9], which necessitates for a rapid and accurate detection system. This is to provide for a better control towards the international trading of agarwood and its products. Conventional identification methods such as through wood anatomy cannot be applied to identify agarwood at the species level [14–16]. Several attempts using molecular markers to characterize different *Aquilaria* species have been carried out, but the prerequisite of a large sampling to serve as a reference database has often limited its application for accurate identification [17–19].

For the last decade, DNA barcoding has been gaining popularity as a rapid, accurate, and convenient method for species identification. Briefly, a DNA barcode is a short DNA sequence that can be used to tell species apart [20]. In the animal kingdom, the mitochondrial gene cytochrome *c* oxidase I (*CO1*) is widely accepted as a universal DNA barcode for almost all species and has been evaluated in amphibians [21], birds [22], fishes [23], insects [24], and mammals [25]. Unfortunately, no single-locus universal DNA barcode has been found capable of resolving the plant kingdom adequately. The *CO1* gene was reportedly unsuitable for higher plants due to the low mutation rate of plant mitochondrial DNA, leading to the suggestions of using chloroplast (cpDNA) and nuclear DNA (nDNA) regions as alternatives. The Consortium for the Barcode of Life (CBOL) proposed a combination of both the cpDNA maturase K (*mat*K) gene and the ribulose-bisphosphate carboxylase (*rbc*L) gene as the core of DNA barcode for plants, especially for angiosperms [26], and further combined them with the non-coding cpDNA *psb*A-*trn*H intergenic spacer [27] and the nuclear ribosomal internal transcribed spacer (ITS) [28] or ITS2 [29] regions to attain high discrimination at the species level. Proposed plant DNA barcode loci were assessed based on their recoverability, sequence quality and levels of species discrimination [26]. Based on several studies on plants, DNA barcodes using a combination of several loci have shown greater discrimination power compared to single-locus barcodes [30]. However in certain cases, single-locus barcodes such as the ITS region was reported to still be able to provide sufficient information for phylogeny construction and species determination, and at the same time providing better resolution in species identification [31,32]. Currently, DNA barcoding is acknowledged as an effective tool for species-level identification in plants, and has contributed in the resolution of relationships among taxa, forensic identification, and species authentication, especially for endangered species and medicinal plants [33–35].

The search for a suitable barcode for Thymelaeaceae was first attempted in 2002 by using a combination of the cpDNA *rbc*L gene and the non-coding intergenic spacer region *trn*L-*trn*F [36]. The barcode was further strengthened by including ITS into the combination, which successfully resolved the family Thymelaeaceae at the tribe level [37]. The reliability of DNA barcoding by applying the *trn*L-*trn*F and ITS regions was first shown using a xylarium specimen of *A*. *sinensis* and was proven applicable on *Aquilaria* wood samples [16]. Indeed, DNA barcoding using wood samples as the source of genomic DNA has been shown to be quite promising [38]. However, a complete DNA sequence database must first be established as a reference prior to adopting DNA barcoding for wood species identification and forensic application.

In this study, we evaluate a subset of eight proposed plant DNA barcode loci for their potential in identifying individual *Aquilaria* species. We also propose and demonstrate the use of a DNA barcode consisting of a combination of loci as a rapid tool to identify the source species of agarwood specimens such as wood chips, wood blocks, and other consumer products. The outcome of this study provides an identification technique for agarwood-producing species, which can be used in timber trade controls and international agarwood trade markets.

## Materials and Methods

### Ethics statement

Samples for use as reference in the form of leaves were collected from individual planted trees in various arboreta in Malaysia or donated by researchers in the respective countries; therefore, they do not require special permits. For arboreta samples, approvals to collect were obtained from the Forestry Faculty of Universiti Putra Malaysia (UPM), and the Forest Research Institute of Malaysia (FRIM). Foreign samples were donated by Centre for Conservation and Rehabilitation of Forestry Research and Development Agency (FORDA), Indonesia, and Institute of Medicinal Plant Development (IMPLAD) of Hainan Branch, China. Other samples were purchased and/or donated by local tree nurseries belonging to the respective Forestry Departments. Localities of all sampled accessions are shown in Table 1.

**Table 1:** Localities, voucher details and GenBank accession numbers of the reference species generated through this study.

Species (Sample number)	Collector’s name and collection number	Region of origin (number of individuals examined)	Sampling location	GenBank accession numbers							
				*mat*K	*rbc*L	*rpo*B	*rpo*C1	*psb*A-*trn*H	*trn*L-*trn*F	ITS	ITS2
*Aquilaria crassna* (1–3)	Mohamed, FBL01012-FBL01014	Vietnam (3)	FRIM Arboretum	KU244186, KU244187, KU244188	KU244212, KU244213, KU244214	KU244134, KU244135, KU244136	KU244160, KU244161, KU244162	KU244056, KU244057, KU244058	KU244030, KU244031, KU244032	KU244082, KU244083, KU244084	KU244108, KU244109, KU244110
*Aquilaria crassna* (4)	Lee & Mohamed, FBL01017	Vietnam (1)	FORDA Arboretum	KU244189	KU244215	KU244137	KU244163	KU244059	KU244033	KU244085	KU244111
*Aquilaria hirta* (1–3)	Lee & Mohamed, FBL01004-FBL01006	Terengganu, Malaysia (3)	Nursery at Forestry Training Center, Chalok, Terengganu,	KU244190, KU244191, KU244192	KU244216, KU244217, KU244218	KU244138, KU244139, KU244140	KU244164, KU244165, KU244166	KU244060, KU244061, KU244062	KU244034, KU244035, KU244036	KU244086, KU244087, KU244088	KU244112, KU244113, KU244114
*Aquilaria malaccensis* (1–3)	Lee & Mohamed, FBL01001- FBL01003	Pahang, Malaysia (3)	Center for Seed and Planting Material Procurement, Lentang, Pahang	KU244193, KU244194, KU244195	KU244219, KU244220, KU244221	KU244141, KU244142, KU244143	KU244167, KU244168, KU244169	KU244063, KU244064, KU244065	KU244037, KU244038, KU244039	KU244089, KU244090, KU244091	KU244115, KU244116, KU244117
*Aquilaria microcarpa* (1–3)	Lee & Mohamed, FBL01018- FBL01020	Kalimantan, Indonesia (3)	FORDA Arboretum	KU244196, KU244197, KU244198	KU244222, KU244223, KU244224	KU244144, KU244145, KU244146	KU244170, KU244171, KU244172	KU244066, KU244067, KU244068	KU244040, KU244041, KU244042	KU244092, KU244093, KU244094	KU244118, KU244119, KU244120
*Aquilaria sinensis* (1–3)	Mohamed, FBL01009-FBL01011	China (3)	FRIM Arboretum	KU244199, KU244200, KU244201	KU244225, KU244226, KU244227	KU244147, KU244148, KU244149	KU244173, KU244174, KU244175	KU244069, KU244070, KU244071	KU244043, KU244044, KU244045	KU244095, KU244096, KU244097	KU244121, KU244122, KU244123
*Aquilaria sinensis* (4–6)	Lee & Mohamed, FBL01021-FBL01023	Hainan, China (3)	Medicinal Plant Garden, Xinglong, IMPLAD Hainan	KU244202, KU244203, KU244204	KU244228, KU244229, KU244230	KU244150, KU244151, KU244152	KU244176, KU244177, KU244178	KU244072, KU244073, KU244074	KU244046, KU244047, KU244048	KU244098, KU244099, KU244100	KU244124, KU244125, KU244126
*Aquilaria subintegra* (1–2)	Mohamed, FBL01015-FBL01016	Thailand (2)	FRIM Arboretum	KU244205, KU244206	KU244231, KU244232	KU244153, KU244154	KU244179, KU244180	KU244075, KU244076	KU244049, KU244050	KU244101, KU244102	KU244127, KU244128
*Aquilaria yunnanensis* (1–3)	Lee & Mohamed, FBL01024-FBL01026	Yunnan, China (3)	Medicinal Plant Garden, Xinglong, IMPLAD Hainan	KU244207, KU244208, KU244209	KU244233, KU244234, KU244235	KU244155, KU244156, KU244157	KU244181, KU244182, KU244183	KU244077, KU244078, KU244079	KU244051, KU244052, KU244053	KU244103, KU244104, KU244105	KU244129, KU244130, KU244131
*Gyrinops versteegii*	Lee & Mohamed, FBL01027	Lombok Island, Indonesia (1)	FORDA Arboretum	KU244210	KU244236	KU244158	KU244184	KU244080	KU244054	KU244106	KU244132
*Gonystylus bancanus*	Lee, FBL01031	Selangor, Malaysia (1)	UPM Arboretum, Ayer Hitam Forest Reserve, Puchong	KU244211	KU244237	KU244159	KU244185	KU244081	KU244055	KU244107	KU244133

FRIM, Forest Research Institute of Malaysia; FORDA, Centre for Conservation and Rehabilitation, Forestry Research and Development Agency, Indonesia; IMPLAD, Institute of Medicinal Plant Development, China; UPM, Universiti Putra Malaysia.

### Plant materials and samples of agarwood products

To test the performance of candidate DNA barcoding loci, a total of seven *Aquilaria* species were selected for use as reference species. Species included are those widely associated with the international agarwood trade market. Agarwood-producing species from two other genera under Thymelaeaceae, *Gyrinops versteegii* and *Gonystylus bancanus* were included to serve as outgroups. Samples in the form of fresh leaves were collected from individual trees in official arboretums, ex-situ botanical gardens, and in-situ nurseries for use in genomic DNA extraction. In some cases, leaves were initially dried and transported back to the laboratory for genomic DNA extraction. Voucher specimens were deposited at the Forest Biotechnology Laboratory in Universiti Putra Malaysia (UPM). DNA sequences generated from these species samples (hereinafter ‘reference samples’) were used as references for the identification of commercial test samples detailed below. Details on the collected reference samples are listed in Table 1.

**Fig 1:**
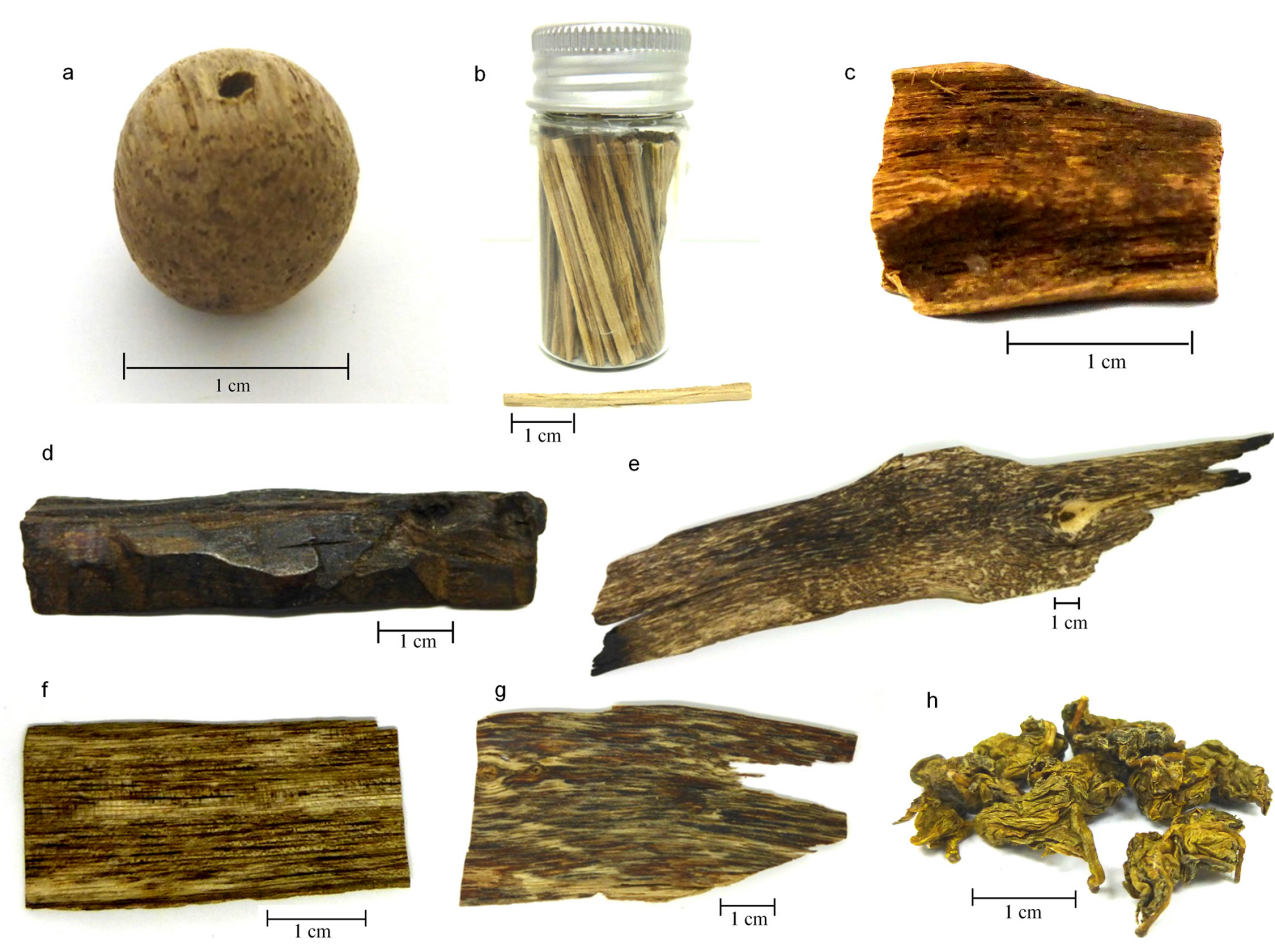
Test samples used in this study. (a) Bead (BD), (b) Cigarette stick (CS), (c) Wood block 1 (WB1), (d) Wood block 2 (WB2), (e) Wood chip 1 (WC1), (f) Wood chip 2 (WC2), (g) Wood chip 3 (WC3), and (h) Processed leaf (PL).

For the purpose of testing the efficacy of the proposed DNA barcode, we also included commercial agarwood samples as case-study samples (hereinafter ‘test samples’). These agarwood samples were obtained from various sources such as through direct purchasing, agarwood-processing factories, and individual collectors. To avoid bias, the purpose of the sample collection and purchases was not revealed to the sellers and for the same reason we did not pursue the origin of the agarwood specimens when they were first obtained. A total of five types of agarwood products were collected: beads (BD), cigarette stick (CS), wood block (WB), wood chip (WC), and processed leaf (PL) (Fig 1). Details on the test samples are given in Table 2.

**Table 2:** List of the agarwood samples tested in this study.

Sample name	Sample form	Seller’s/ Collector’s location	Claimed region/ country of origin	Claimed species of origin	Trade name	GenBank accession for proposed DNA barcode		Species of origin identified through the proposed DNA barcode (*trn*L-*trn*F+ITS2)
						*trn*L-*trn*F	ITS2	
BD	Bead	Penang, Malaysia	Unknown	Unknown	Imported agarwood	KU238024	KU238032	*Aquilaria malaccensis*
CS	Cigarette stick	Guangdong, China	Hainan, China	*A*. *sinensis*	Hainanese agarwood	KU238026	KU238034	*Aquilaria sinensis*
WB1	Wood block	Guangdong, China	Kalimantan, Indonesia	Unknown	Indonesian agarwood	KU238025	KU238033	*Aquilaria malaccensis*
WB2	Wood block	Kuching, Malaysia	Kalimantan, Indonesia	Unknown	Gaharu	KU238028	KU238036	*Aquilaria malaccensis*
WC1	Wood chip	Penang, Malaysia	Vietnam	*A*. *crassna*	Vietnamese agarwood	KU238027	KU238035	*Aquilaria crassna*
WC2	Wood chip	Guangdong, China	Nha Trang, Vietnam	Unknown	Vietnamese agarwood	KU238029	KU238037	*Aquilaria crassna*
WC3	Wood chip	Guangdong, China	Laos	Unknown	Lao agarwood	KU238030	KU238038	*Aquilaria crassna*
PL	Processed leaf (*Aquilaria* tea)	Hainan, China	Guangdong, China	*A*. *sinensis*	Chinese agarwood	KU238031	KU238039	*Aquilaria sinensis*

### DNA isolation

For fresh leaf and processed leaf samples, 100 mg of leaf material was pulverized in liquid nitrogen using mortar and pestle. Genomic DNA was then extracted using the DNeasy Plant Mini kit (Qiagen, Germany) following manufacturer’s recommendations. For wood samples, a slight modification to the manufacturer’s protocol was applied, as suggested by [16]. Beads, cigarette stick, wood blocks, and wood chips were first sliced into small slender strips using a sterile scalpel and a total of 100 mg of the sliced wood was then inserted into a 2 ml-microcentrifuge tube containing 1000 μl Buffer AP1, 1% (w/v) polyvinylpyrolidone (PVP), and 8 μl RNase A, followed by incubation at 65°C for 6 hours. Then the sample was let to cool to room temperature and 280 μl Buffer P3 was added before further incubation at -20°C for 2 hours. Subsequent steps were as manufacturer’s protocol. The quantity and quality of extracted DNA were determined by spectrophotometry (Nanophotometer, IMPLEN, USA).

### PCR amplification and DNA sequencing

A total of eight candidate DNA barcode loci were amplified and sequenced from the total genomic DNA of the reference samples. Established primers were used to amplify four coding cpDNA loci, *mat*K, *rbc*L, *rpo*B, and *rpo*C1, two non-coding cpDNA intergenic spacer loci, *psb*A-*trn*H and *trn*L-*trn*F, and the nDNA loci, ITS and ITS2. For agarwood samples, the *trn*L-*trn*F locus was amplified using additional internal primers: 1) primer e was coupled with the primer E-Aq-rev-1, and 2) primer f was coupled with primer F-forw-2 [17]. Details on the primers are listed in Table 3. PCR was conducted in a final reaction volume of 25 μL, containing 12.5 μL of 2x PCRBIO Taq Mix Red (PCRBiosystems, UK), 10 mM of each primer, and 25 ng of genomic DNA as template. PCR amplification was conducted on a SpeedCycler^2^ Thermal Cycler (Analytik Jena, Germany). Successful PCR amplification was inspected through electrophoresis on 1% agarose gel, before DNA sequencing on an ABI PRISM 3730xl Genetic Analyzer (Applied Biosystems, USA).

**Table 3:** Details on the PCR primers used in this study.

DNA barcode locus	Primer	Primer sequence (5’-3’)	References
*mat*K	3F_KIM	CGTACAGTACTTTTGTGTTTACGAG	Kim, unpublished
	1R_KIM	ACCCAGTCCATCTGGAAATCTTGGTTC	Kim, unpublished
*rbc*L	a_F	ATGTCACCACAAACAGAGACTAAAGC	[27]
	a_R	CTTCTGCTACAAATAAGAATCGATCTC	[27]
*rpo*B	1f	AAGTGCATTGTTGGAACTGG	[39]
	4r	GATCCCAGCATCACAATTCC	[39]
*rpo*C1	2f	GGCAAAGAGGGAAGATTTCG	[39]
	4r	CCATAAGCATATCTTGAGTTGG	[39]
*psb*A-*trn*H	psbA3-f	GTTATGCATGAACGTAATGCTC	[40]
	trnHf_05	CGCGCATGGTGGATTCACAATCC	[41]
*trn*L-*trn*F	e	GGTTCAAGTCCCTCTATCCC	[42]
	f	ATTTGAACTGGTGACACGAG	[42]
	E-Aq-rev-1	CGAACGGGAATTGACAGAAT	[17]
	F-forw-2	CAAATCAACATTTTTGAGTAAGGAA	[17]
ITS	ITS92	AAGGTTTCCGTAGGTGAAC	[43]
	ITS75	TATGCTTAAACTCAGCGGG	[43]
ITS2	ITS-S2F	ATGCGATACTTGGTGTGAAT	[44]
	ITS-S3R	GACGCTTCTCCAGACTACAAT	[44]

### Additional sequences

We downloaded additional sequences (of loci *mat*K, *rbc*L, *rpo*B, *rpo*C1, *psb*A-*trn*H, *trn*L-*trn*F, ITS, and ITS2) belonging to *Aquilaria* from NCBI and added to the list of sequences we generated in this study. We selected sequences that are over 300 bp in length and of known species identity. Because DNA sequence information for *Aquilaria* in the GenBank database is limited, sequences not generated from voucher specimens were included as well. The taxa and the corresponding GenBank accession numbers of the sequences used in this study are shown in Table 4.

**Table 4:** Barcode sequences downloaded from NCBI GenBank and sequences generated from this study and used in TaxonDNA analysis. Underlined GenBank accession numbers indicate the sequences generated from this study.

DNA barcode locus	Species	GenBank accession
*mat*K	*A*. *beccariana*	FJ572802
	*A*. *crassna*	KU244186, KU244187, KU244188, KU244189
	*A*. *hirta*	KU244190, KU244191, KU244192
	*A*. *malaccensis*	KJ499918, KJ499942, KJ499949, KU244193, KU244194, KU244195
	*A*. *microcarpa*	KU244196, KU244197, KU244198
	*A*. *sinensis*	HQ415244, KP093250, KP093251, KR530384, KR530385, KU244199, KU244200, KU244201, KU244202, KU244203, KU244204
	*A*. *subintegra*	KU244205, KU244206
	*A*. *yunnanensis*	KR580386, KR530387, KR530388, KU244207, KU244208, KU244209
*rbc*L	*A*. *beccariana*	Y15149
	*A*. *crassna*	KU244212, KU244213, KU244214, KU244215
	*A*. *hirta*	KU244216, KU244217, KU244218
	*A*. *malaccensis*	KJ667626, KJ667670. KU244219, KU244220, KU244221
	*A*. *microcarpa*	KU244222, KU244223, KU244224
	*A*. *sinensis*	GQ436619, GQ436620, KP094157, KP094158, KR528751, KR528752, KU244225, KU244226, KU244227, KU244228, KU244229, KU244230
	*A*. *subintegra*	KU244231, KU244232
	*A*. *yunnanensis*	KR528753, KR528754, KR528755, KR528756, KU244233, KU244234, KU244235
*rpo*B	*A*. *crassna*	KU244134, KU244135, KU244136, KU244137
	*A*. *hirta*	KU244138, KU244139, KU244140
	*A*. *malaccensis*	KU244141, KU244142, KU244143
	*A*. *microcarpa*	KU244144, KU244145, KU244146
	*A*. *sinensis*	KU244147, KU244148, KU244149, KU244150, KU244151, KU244152
	*A*. *subintegra*	KU244153, KU244154
	*A*. *yunnanensis*	KU244155, KU244156, KU244157
*rpo*C1	*A*. *crassna*	KU244160, KU244161, KU244162, KU244163
	*A*. *hirta*	KU244164, KU244165, KU244166
	*A*. *malaccensis*	KJ749922, KJ749925, KJ749934, KU244167, KU244168, KU244169
	*A*. *microcarpa*	KU244170, KU244171, KU244172
	*A*. *sinensis*	KU244173, KU244174, KU244175, KU244176, KU244177, KU244178
	*A*. *subintegra*	KU244179, KU244180
	*A*. *yunnanensis*	KU244181, KU244182, KU244183
*psb*A-*trn*H	*A*. *crassna*	KU244056, KU244057, KU244058, KU244059
	*A*. *hirta*	KU244060, KU244061, KU244062
	*A*. *malaccensis*	KU244063, KU244064, KU244065
	*A*. *microcarpa*	KU244066, KU244067, KU244068
	*A*. *sinensis*	GQ435290, GQ435291, HQ415408, KM668558, KM668559, KM668560, KP095710, KP095711 KR533790, KR533792, KU244069, KU244070, KU244071, KU244072, KU244073, KU244074
	*A*. *subintegra*	KU244075, KU244076
	*A*. *yunnanensis*	KR533788, KR533789, KR533791, KR533793, KR533794, KU244077, KU244078, KU244079
*trn*L-*trn*F	*A*. *beccariana*	AY216740, AY216741,
	*A*. *citrinicarpa*	AY216742
	*A*. *crassna*	AY216743, KU244030, KU244031, KU244032, KU244033
	*A*. *filaria*	AY216766
	*A*. *hirta*	KU244034, KU244035, KU244036
	*A*. *khasiana*	AY216744
	*A*. *malaccensis*	AY216745, AY216746, AY216747, KU244037, KU244038, KU244039
	*A*. *microcarpa*	KU244040, KU244041, KU244042
	*A*. *parvifolia*	AY216748
	*A*. *urdanetensis*	AY216750
	*A*. *sinensis*	AY216749, EU652672, EU652673, EU652674, EU652675, EU652676, EU652677, EU652678, EU652679, EU652680, GU736358, KF018041, KU244043, KU244044, KU244045, KU244046, KU244047, KU244048
	*A*. *subintegra*	KU244049, KU244050
	*A*. *yunnanensis*	EU652681, KU244051, KU244052, KU244053
ITS	*A*. *crassna*	AY920326, AY920327, KU244082, KU244083, KU244084, KU244085
	*A*. *hirta*	KU244086, KU244087, KU244088
	*A*. *malaccensis*	KF636365, KM887409, KM887429, KM887433, KU244089, KU244090, KU244091
	*A*. *microcarpa*	KU244092, KU244093, KU244094
	*A*. *rugosa*	AY920328, AY920329, AY920330
	*A sinensis*	EF645833, EF645834, EF645836, FJ980392, GQ891956, KP093005, KP093006, KF636364, KR531769, KU244095, KU244096, KU244097, KU244098, KU244099, KU244100
	*A*. *subintegra*	KU244101, KU244102
	*A*. *yunnanensis*	EF645835, KR531771, KR531772, KR531773, KU244103, KU244104, KU244105
ITS2	*A*. *crassna*	KU244108, KU244109, KU244110, KU244111
	*A*. *hirta*	KU244112, KU244113, KU244114
	*A*. *malaccensis*	KU244115, KU244116, KU244117
	*A*. *microcarpa*	KU244118, KU244119, KU244120
	*A*. *sinensis*	GQ434674, GQ434675, KC441012, KC441013, KJ748403, KJ748404, KJ748405, KJ748406, KJ748407, KJ749408, KJ748409, KM870777, KR531768, KU244121, KU244122, KU244123, KU244124, KU244125, KU244126
	*A*. *subintegra*	KU244127, KU244128
	*A*. *yunnanensis*	KR531770, KU244129, KU244130, KU244131

### Data analysis

DNA sequences generated from this study were assembled and aligned using Gene Runner version 3.05, saved in FASTA format and deposited onto GenBank (Table 1). The eight candidate DNA barcode loci and their combinations were evaluated using three different methods, i.e. (1) genetic distance and barcoding gaps, (2) level of species discrimination, and (3) phylogenetic tree. Genetic distances for both inter- and intra-specific distances were calculated using the Kimura 2-parameter model [45] in MEGA 6 [46]. Barcoding gaps comparing the distributions of the pairwise inter- and intra-specific distances for each candidate and possible combination with 0.005 distance intervals were estimated using the “pairwise summary” function in TaxonDNA [47]. The accuracy in species assignment for each potential DNA barcodes were further calculated using “best match”, “best close match”, and “all species barcodes” functions embedded in TaxonDNA. The effectiveness of the candidate barcodes were then further evaluated through phylogenetic tree-based analysis. Phylogenetic trees were generated using the neighbor-joining (NJ) method in MEGA 6, with individual node support calculated based on 1000 bootstrap re-samplings and all positions containing gaps and missing data were included for analysis (pairwise deletion).

## Results

### PCR amplification and DNA sequencing

PCR amplification and DNA sequencing of all eight DNA barcoding loci were successful for all reference samples. A total of 208 barcode sequences (eight sequences for each individual from a total of 26 individuals) were generated representing the seven selected *Aquilaria* species as well as the outgroup species, *Gyrinops versteegii* and *Gonystylus bancanus*. In addition, a total of 103 sequences from NCBI GenBank database were downloaded, comprising of *mat*K (12), *rbc*L (13), *rpo*C1 (3), *psb*A-*trn*H (15), *trn*L-*trn*F (24), ITS (22), and ITS2 (14) sequences (number in parenthesis represents the number of sequences downloaded for each locus) (Table 4). There is no record of *Aquilaria rpo*B in the GenBank database at the time of manuscript preparation. For the eight test samples, only the best combination DNA barcode (*trn*L-*trn*F+ITS2, discussed in later sections of this article) was sequenced, generating 16 barcode DNA sequences.

### Intra- and inter-specific genetic variation of *Aquilaria*

Combining all reference DNA sequences, the aligned DNA sequence lengths ranged from 441 bp (*psb*A-*trn*H) to 886 bp (*mat*K). ITS had the most variable sites, followed by ITS2 and *mat*K (Table 5). The pairwise intra-specific distances in the eight barcode loci ranged from 0.00% to 0.38% (Table 6). The mean intraspecific distances ranged from 0.00% (*psb*A-*trn*H and *trn*L-*trn*F) to 0.11% (ITS). The pairwise interspecific distances in the eight barcode loci ranged from 0.00% to 3.03%. The mean interspecific distances ranged from 0.05% (*rpo*C1) to 1.60% (ITS). Generally, ITS exhibits the highest mean intra- and inter-specific distance in this study.

**Table 5:** Evaluation of the eight DNA barcode loci.

	DNA barcode locus							
Parameters assessed	*mat*K	*rbc*L	*rpo*B	*rpo*C1	*psb*A-*trn*H	*trn*L-*trn*F	ITS	ITS2
Number of individuals	24	24	24	24	24	24	24	24
PCR success (%)	100	100	100	100	100	100	100	100
Sequencing success (%)	100	100	100	100	100	100	100	100
Sequence length	885–886	644	512	529	441	465–471	683–685	496–497
Aligned length	886	644	512	529	441	471	685	497
No. of variable sites	9	6	4	3	1	7	31	14
No. of indels	0	0	0	0	0	6	3	1

**Table 6:** Genetic distance percentage generated using Kimura 2-parameter model analysis for the candidate barcode loci and their combinations.

	Intraspecific distance (%)			Interspecific distance (%)		
Barcode loci and combinations	Minimum	Maximum	Mean	Minimum	Maximum	Mean
a) *mat*K	0.00	0.07	0.01	0.00	0.85	0.39
b) *rbc*L	0.00	0.31	0.05	0.00	0.47	0.15
c) *rpo*B	0.00	0.26	0.07	0.00	0.26	0.07
d) *rpo*C1	0.00	0.38	0.06	0.00	0.19	0.05
e) *psb*A-*trn*H	0.00	0.00	0.00	0.00	0.23	0.13
f) *trn*L-*trn*F	0.00	0.00	0.00	0.00	0.87	0.60
g) ITS	0.00	0.23	0.11	0.00	3.03	1.60
h) ITS2	0.00	0.13	0.03	0.00	2.05	1.09
i) *trn*L-*trn*F+ITS	0.00	0.13	0.07	0.04	2.11	1.19
j) *trn*L-*trn*F+ITS2	0.00	0.07	0.02	0.00	1.47	0.85
k) *trn*L-*trn*F+*psb*A-*trn*H	0.00	0.07	0.01	0.00	0.55	0.37
l) *trn*L-*trn*F+*psb*A-*trn*H+ITS	0.00	0.10	0.05	0.03	1.60	0.89
m) *trn*L-*trn*F+*psb*A-*trn*H+ITS2	0.00	0.10	0.02	0.00	1.08	0.62
n) *mat*K+*trn*L-*trn*F	0.00	0.04	0.01	0.00	0.78	0.46
o) *mat*K+*rbc*L+*trn*L-*trn*F	0.00	0.10	0.02	0.00	0.63	0.36
p) *mat*K+ITS	0.00	0.14	0.05	0.03	1.72	0.91
q) *mat*K+ITS2	0.00	0.08	0.02	0.00	1.18	0.64
r) *mat*K+*rbc*L+ITS	0.00	0.10	0.06	0.00	1.23	0.69
s) *mat*K+*rbc*L+ITS2	0.00	0.13	0.03	0.00	0.92	0.48
t) *mat*K+*trn*L-*trn*F+ITS	0.00	0.10	0.04	0.02	1.52	0.94
u) *mat*K+*trn*L-*trn*F+ITS2	0.00	0.06	0.02	0.00	1.10	0.63
v) *mat*K+*rbc*L+*trn*L-*trn*F+ITS	0.00	0.08	0.05	0.02	1.17	0.67
w) *mat*K+*rbc*L+*trn*L-*trn*F+ITS2	0.00	0.11	0.03	0.00	0.87	0.50
x) *mat*K*+rbc*L	0.00	0.13	0.03	0.00	0.62	0.29
y) *rbc*L*+trn*L*-trn*F+ITS	0.00	0.11	0.06	0.00	1.42	0.81

### Barcoding gap test

The barcoding gaps between intra- and inter-specific distances were evaluated by constructing the distribution graph from the results obtained in the “pairwise summary” function in TaxonDNA. No single- or multi-locus barcode displayed clear barcoding gaps; all of them overlapped between the intra- and inter-specific distances. Distribution graphs computed are shown in S1 Fig.

### Species discrimination

TaxonDNA was used to analyze all sequences generated in this study as well as those downloaded from the GenBank database (Table 7). Based on the analysis for “best match” and “best close match”, *mat*K, *rbc*L, *psb*A-*trn*H, *trn*L-*trn*F, ITS, and ITS2 each provided species identification for 33.33, 13.51, 8.10, 58.33, 46.80, and 42.85% of the reference samples, respectively. However, species identification was zero for both *rpo*B and *rpo*C1 in this study. As for the “all species barcodes” analysis, most of the candidate barcodes had higher percentages than the “best match” and “best close match” analyses, except for *trn*L-*trn*F and ITS. For single-locus barcodes, *trn*L-*trn*F turned out to have the highest success rate among the eight potential DNA barcode loci. However, loci combinations (LC) that included ITS provided higher success rates than other combination barcodes. The highest success rate was obtained by combining *trn*L-*trn*F with ITS (75.00%) or ITS2 (75.00%), with the latter having a higher “all sequence barcode” percentage (LC-i, *trn*L-*trn*F+ITS = 66.66%; LC-j, *trn*L-*trn*F+ITS2 = 79.16%).

**Table 7:** Species identification success rate based on TaxonDNA analysis

Barcode loci and combinations	Best match (%)	Best close match (%)	All species barcodes (%)
a) *mat*K	33.33	33.33	44.44
b) *rbc*L	13.51	13.51	72.97
c) *rpo*B	0.00	0.00	66.66
d) *rpo*C1	0.00	0.00	70.37
e) *psb*A-*trn*H	8.10	8.10	72.97
f) *trn*L-*trn*F	58.33	58.33	14.58
g) ITS	46.80	46.80	40.42
h) ITS2	42.85	42.85	60.71
i) *trn*L-*trn*F+ITS	75.00	75.00	66.66
j) *trn*L-*trn*F+ITS2	75.00	75.00	79.16
k) *trn*L-*trn*F+*psb*A-*trn*H	50.00	50.00	66.66
l) *trn*L-*trn*F+*psb*A-*trn*H+ITS	69.23	69.23	61.53
m) *trn*L-*trn*F+*psb*A-*trn*H+ITS2	75.00	75.00	79.16
n) *mat*K+*trn*L-*trn*F	66.66	66.66	50.00
o) *mat*K+*rbc*L+*trn*L-*trn*F	61.53	61.53	46.15
p) *mat*K+ITS	70.83	70.83	79.16
q) *mat*K+ITS2	66.66	66.66	75.00
r) *mat*K+*rbc*L+ITS	70.83	70.83	73.07
s) *mat*K+*rbc*L+ITS2	66.66	66.66	75.00
t) *mat*K+*trn*L-*trn*F+ITS	70.83	70.83	50.00
u) *mat*K+*trn*L-*trn*F+ITS2	65.38	65.38	61.53
v) *mat*K+*rbc*L+*trn*L-*trn*F+ITS	70.83	70.83	54.16
w) *mat*K+*rbc*L+*trn*L-*trn*F+ITS2	70.83	70.83	66.66
x) *mat*K*+rbc*L	66.66	66.66	75.00
y) *rbc*L*+trn*L*-trn*F+ITS	75.00	75.00	66.66

Note: Description on query identification criteria for “best match”, best close match” and “all species barcodes” is based on [47].

### Phylogenetic tree analysis

**Fig 2:**
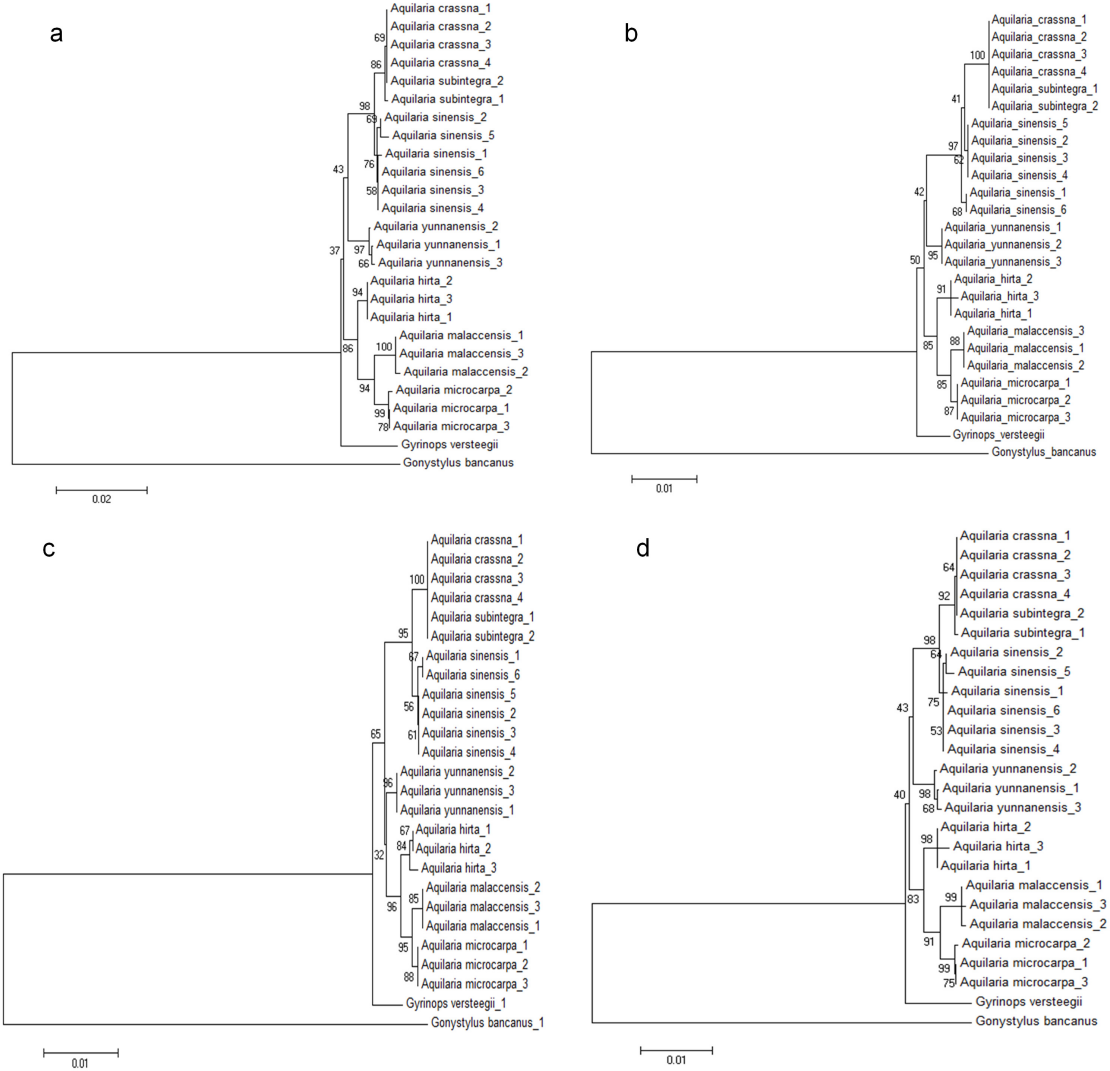
Neighbor-joining trees constructed from combination markers that yielded high species resolution identified from TaxonDNA. (a) *trn*L-*trn*F+ITS, (b) *trn*L-*trn*F+ITS2, (c) *trn*L-*trn*F+*psb*A-*trn*H+ITS2, (d) *rbc*L+*trn*L-*trn*F+ITS.

To put the candidate DNA barcodes evaluated by TaxonDNA into perspective, candidate barcode loci and their combinations that gave the highest percentage in species resolution (best match and best close match of 75%) from the TaxonDNA analysis (Table 7) were selected for constructing NJ trees. They were LC-i (*trn*L-*trn*F+ITS), LC-j (*trn*L-*trn*F+ITS2), LC-m (*trn*L-*trn*F+*psb*A-*trn*H+ITS2), and LC-y (*rbc*L+*trn*L-*trn*F+ITS). All the four NJ trees (Fig 2a–2d) displayed similar clustering patterns. In general, these barcode combinations were able to resolve members of the genus *Aquilaria* by clustering every species into separate clades, except for *A*. *crassna* and *A*. *subintegra*, which had very similar sequences for all the barcode loci.

### Species identification of agarwood samples

**Fig 3:**
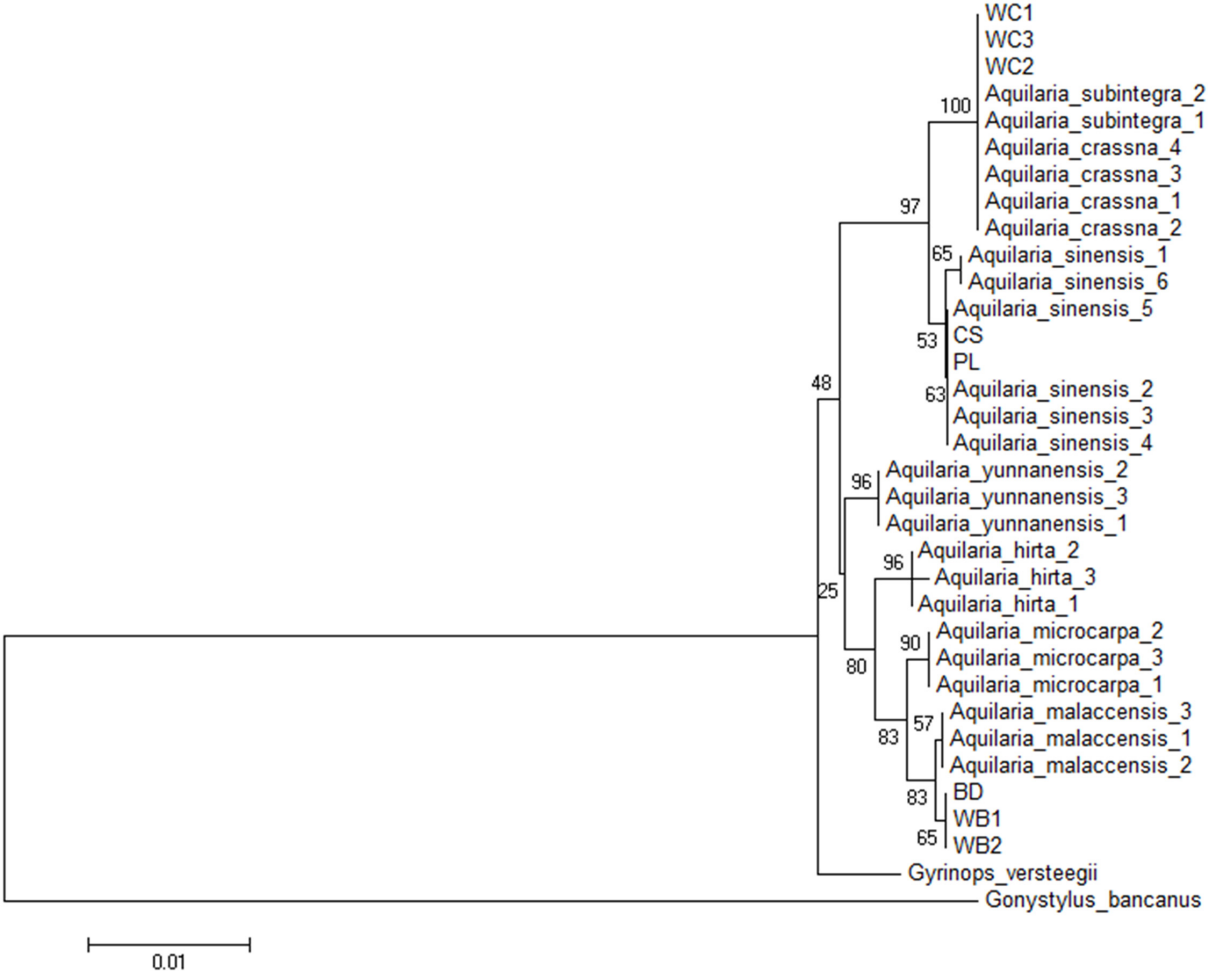
Neighbor-joining tree constructed using *trn*L-*trn*F+ITS2 for agarwood origin identification.

By adopting the proposed best combination LC-j *trn*L-*trn*F+ITS2 barcode as revealed from this study, we set to identify species origin of the eight agarwood test samples (Table 2). Using the NJ tree constructed from barcode sequences of the reference and test samples (Fig 3), we identified the bead (BD) and wood block (WB1 and WB2) samples as closest to *A*. *malaccensis*. Meanwhile, the woodchips had a 100% match to *A*. *crassna* and *A*. *subintegra*. The cigarette stick and tea, which are highly processed products, came out to be *A*. *sinensis*.

## Discussion

### Evaluation of *Aquilaria* DNA barcodes

The concept of an ideal DNA barcode is that it is short (300–800 bp) making it easy for PCR amplification, contains sufficient information to differentiate among a large dataset, and being able to discriminate at the species level [20]. For plants, no single barcode locus has been found to be able to distinguish the whole plant kingdom. The combination of *mat*K+*rbc*L (LC-x) as proposed by CBOL appears as most suitable for discriminating plant taxa at the genus level at best [26]. In previous studies, the combination barcode of two cpDNA loci, *rbc*L+*trn*L-*trn*F, has been proposed for the Thymelaeaceae family [36], but was found to be inconsistent at the tribe level [48,49]. Later, the nDNA ITS locus was also introduced into the analysis. The *rbc*L+*trn*L-*trn*F+ITS (LC-y) combination is shown to provide an accurate species resolution at the tribe level [37]. Thus, it has clearly demonstrated that DNA barcodes using only one or more cpDNA loci was insufficient to provide resolution for Thymelaeaceae, and the addition of the ITS locus seemed useful [36,37]. From this study, although loci from both cpDNA and nDNA exhibit high success rates in PCR amplification and sequencing, single or combination cpDNA barcode loci demonstrated lower discrimination power (<67%) in resolving *Aquilaria* species compared to when combined with nDNA loci like ITS and ITS2 (>65%) (Table 7). The ITS and ITS2 loci contained higher numbers of variable sites compared to the other six single-locus cpDNA barcode loci. Although both the ITS and ITS2 loci showed average intra-specific variation, the inter-specific divergence in *Aquilaria* was high (Table 6); thus, supporting its high efficiency in discriminating closely related species. However, in the case of a single-locus barcode for species identification, *trn*L-*trn*F could resolve more species when compared to any of the ITS sequences (Table 7). The former is able to resolve phylogenetic relationship of the Aquilarieae tribe [17], while the latter is informative at identifying genetic variation among different populations or between closely related *Aquilaria* species from the same geographical region [50–53]. Distinct increase in the successful identification rate for “all species barcodes” for single locus barcodes in this study (*rbc*L, *rpo*B, *rpo*C1, *psb*A-*trn*H) was observed (Table 7). Among all three approaches (best match”, “best close match”, and “all species barcodes”, the “all species barcodes is known as the strictest one, providing no identification if query sequence matches were found to be below the proposed threshold. However queries were considered as successfully identified when matched with at least two conspecific barcodes of the species in question [47]. Therefore its identification criterion explains the distinct increment in successful identification compared to the “best match” and “best close match” approaches, among *Aquilaria* species in this study. The same observation was made in a study on the DNA barcoding of *Gossypium* [54].

The barcode proposed by Rauntenbach [37], i.e. the combination barcode *rbc*L+*trn*L-*trn*F+ITS (LC-y), had “all species barcodes” results identical to the *trn*L-*trn*F+ITS (LC-i) combination as we demonstrated in this study (Table 7). This makes the addition of *rbc*L in species resolution for *Aquilaria* unnecessary. On the other hand, the core barcode *mat*K+*rbc*L (LC-x) as proposed by CBOL had species resolution percentage (“best match” and “best close match”) of 66.66%, making it less suitable to be considered as a useful barcode. In this study, the four best combination loci (LC-i, -j, -m, and -y) achieved species resolution percentage of 75.00%. We propose the LC-i (*trn*L-*trn*F+ITS2) as the best combination DNA barcode to potentially resolve species identity in this genus because: (1) it has the highest percentage in the “all species barcodes” (79.16%) as revealed by TaxonDNA, (2) the ITS2 sequence is relatively short (~500 bp), which makes it amenable to PCR amplification and sequencing even for degraded samples, and (3) the barcode is more cost- and time-efficient because it is a combination of only two loci, compared to the three-locus combination (*trn*L-*trn*F+*psb*A-*trn*H+ITS2 and *rbc*L+*trn*L-*trn*F+ITS). It is worth mentioning here that the DNA barcodes evaluated in this study were not adequate to resolve the identities of *A*. *crassna* and *A*. *subintegra*. This was supported by the result obtained from TaxonDNA using the combination loci *trn*L-*trn*F+ITS2. The percentage in TaxonDNA was evaluated based on successful identification upon individuals included, whereby 18 individuals out of 24 individuals were successful identified (i.e. 18/24 = 75%); 6 individuals (4 from *A*. *crassna* and 2 from *A*. *subintegra*) were ambiguous (data not shown). This is because the DNA sequences of these two species are highly similar, suggesting a lack of genetic divergence between the two species, although they are identifiable through different morphological characteristics [55].

### Geographical clustering

**Fig 4:**
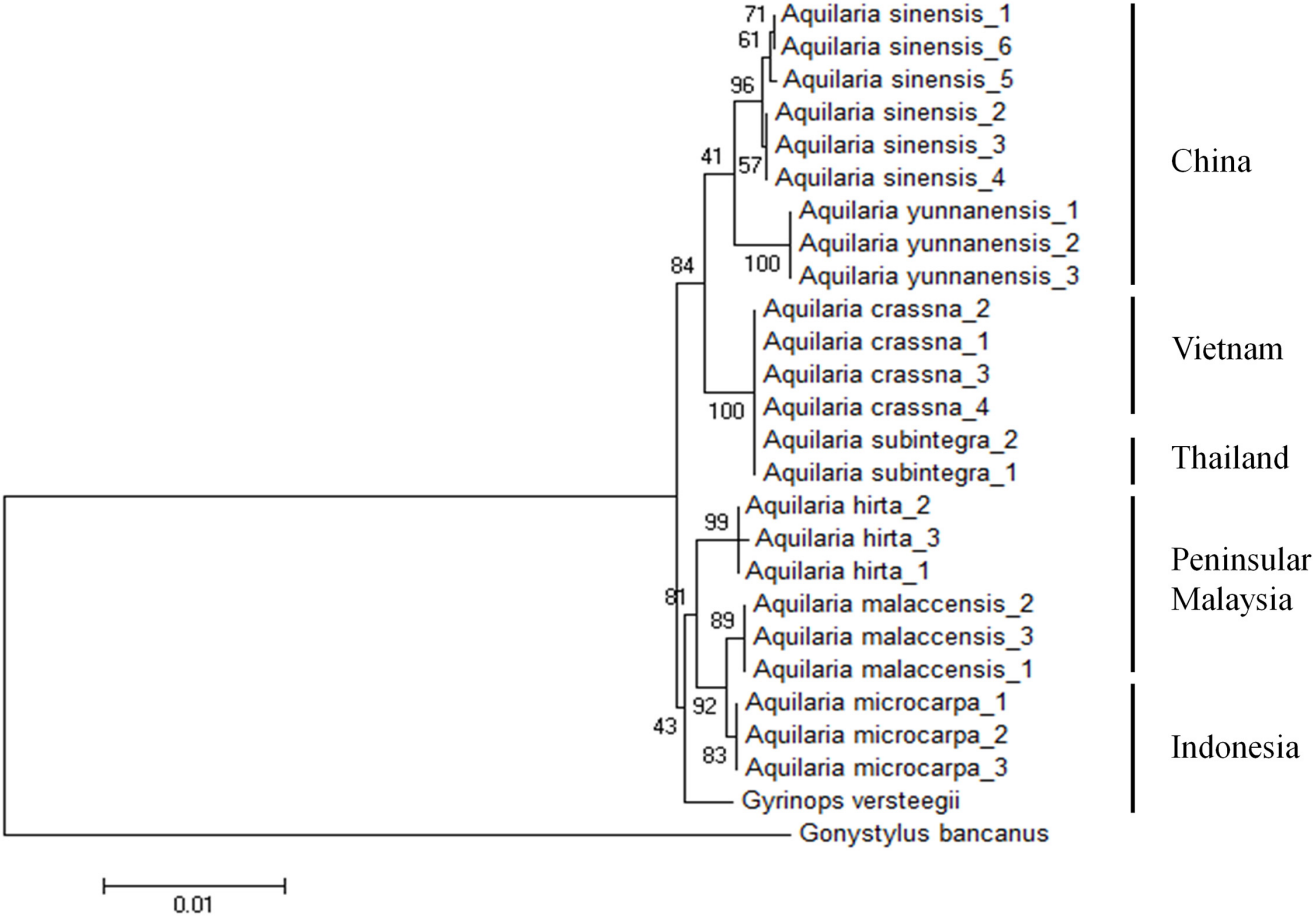
Neighbor-joining tree constructed using *mat*K+*trn*L-*trn*F+ITS2 showing geographical clustering pattern in *Aquilaria* species.

The addition of *mat*K in our proposed combination DNA barcode (LC-u, *mat*K+*trn*L-*trn*F+ITS2) yielded a lower percentage in species resolution when compared to other combination markers, however, interestingly it has the ability to cluster *Aquilaria* species according to their geographical origins. Two species from China, *A*. *sinensis* and *A*. *yunnanensis* are clustered together, followed by a clade representing the Indochina species comprising of *A*. *crassna* (Vietnam) and *A*. *subintegra* (Thailand), and a separate clade for species in the Malesian region: *A*. *malaccensis* and *A*. *hirta* from the Malay Peninsula, and *A*. *microcarpa* from Kalimantan on the Borneo island (Fig 4).

### DNA barcoding in identifying agarwood origin

Efforts to identify the source species of agarwood and its products using molecular tools have been carried out previously: Eurlings and Gravendeel [17] showed the possibility to identify confiscated agarwood products using the *trn*L-*trn*F locus, but only three out of five wood chip samples yielded genomic DNA that was adequate for DNA sequencing. Mohamed et al. [56] utilized a part of the *trn*L-*trn*F region to identify processed agarwood products using real-time PCR technique. However, both reported identification at the genus level, reflecting a clear limitation of the single-locus barcode, *trn*L-*trn*F. Jiao et al. [16] optimized the DNA extraction protocol for *Aquilaria* wood samples and suggested that DNA barcoding is applicable in agarwood species identification. However, a suitable DNA barcode must be first agreed upon.

Because the bead and wood block samples were identified closest to *A*. *malaccensis*, we tried to further investigate if they could be sourced from a different *Aquilaria* species. The *trn*L-*trn*F and ITS2 sequences of the three samples were analyzed via BLAST. They turned out to be 99% identical to other *Aquilaria* species (Table 8). When aligned to the *A*. *malaccensis* sequence generated from this study, a site variation was found occurring at the 323-bp position of the *trn*L-*trn*F sequence (guanine, G, for all three samples and thymine, T, for *A*. *malaccensis*), while no variation was found in the ITS2 sequence. While no threshold genetic divergence has been agreed upon for species discrimination [57], we believe that such small amount of genetic variation (<1%) may very well represent intra-specific genetic variation. In the case of the woodchips, despite having 100% sequence similarity to both *A*. *crassna* and *A*. *subintegra*, we think that the three woodchip samples are sourced from *A*. *crassna* rather than *A*. *subintegra*, as the former is widely cultivated in countries such as Myanmar, Laos, Vietnam, and Thailand. *A*. *subintegra* has very limited natural distribution, which is in southern Thailand and are currently planted in scattered areas in Peninsular Malaysia.

**Table 8:** Nucleotide identity match for agarwood sample BD, WB1 and WB2 based on *trn*L-*trn*F and ITS2 using BLAST analysis

DNA marker	Identity match (%)	Species	Accession No.
*trn*L-*trn*F	99	*A*. *beccariana*	AY216740
		*A*. *crassna*	AY216743
		*A*. *citrinicarpa*	AY216742
		*A*. *malaccensis*	AY216746
		*A*. *parvifolia*	AY216748
		*A*. *sinensis*	EU652677
		*A*. *urdantensis*	AY216750
ITS2	99	*A*. *malaccensis*	KF636365
		*A*. *rugosa*	AY920328

Based on our findings, we conclude that the DNA barcoding technique is useful in identifying the species of origin for agarwood products found in the market. Furthermore, it can be used as a tool to identify agarwood adulterants and fake *Aquilaria* products. As mentioned earlier, the classification of agarwood has always been according to their geographical origin/source. By comparing information provided by the agarwood sellers (Table 2), the information on the species of origin detected through DNA barcoding is well-correlated with the geographical region declared by the sample providers.

## Conclusion

We showed in this study that a combination barcode of *trn*L-*trn*F+ITS2 is useful for species discrimination within the *Aquilaria* genus. Using this proposed combination barcode, the taxonomic identity of agarwood products sourced from the market was successfully established. The development of a DNA barcode library for *Aquilaria* is essential to the agarwood industry as to secure the right for consumers to authenticate the market samples and to tackle agarwood-trade frauds in an effective manner. In terms of traditional medicines, DNA barcoding of *Aquilaria* provides a practical procedure to authenticate *Aquilaria*-based drugs, thus reducing undesirable consequences due to the use of the wrong plant material.

## Supporting Information

S1 Fig Distribution of intra- and inter-specific Kimura 2-parameter (K2P) distances among all samples for the eight candidate loci and their combinations. (a) *mat*K, (b) *rbc*L, (c) *rpo*B, (d) *rpo*C1, (e) *psb*A-*trn*H, (f) *trn*L-*trn*F, (g) ITS, (h) ITS2, (i) *trn*L-*trn*F+ITS, (j) *trn*L-*trn*F+ITS2, (k) *trn*L-*trn*F+*psb*A-*trn*H, (l) *trn*L-*trn*F+*psb*A-*trn*H+ITS, (m) *trn*L-*trn*F+*psb*A-*trn*H+ITS2, (n)*mat*K+*trn*L-*trn*F, (o) *mat*K+*rbc*L+*trn*L-*trn*F, (p) *mat*K+ITS, (q) *mat*K+ITS2, (r) *mat*K+*rbc*L+ITS, (s) *mat*K+*rbc*L+ITS2, (t) *mat*K+*trn*L-*trn*F+ITS, (u) *mat*K+*trn*L-*trn*F+ITS2, (v) *mat*K+*rbc*L+*trn*L-*trn*F+ITS, (w) *mat*K+*rbc*L+*trn*L-*trn*F+ITS2, (x) *mat*K+*rbc*L and (y) *rbc*L+*trn*L-*trn*F+ITS
(PDF)

## References

[1] Nobuchi T, Siripatanadilok S. Preliminary observation of *Aquilaria crassna* wood associated with the formation of aloeswood. Bulletin of the Kyoto University Forests. 1991; 63: 226–235.

[2] Pojanagaroon S, Kaewrak C. Mechanical methods to stimulate aloes wood formation in *Aquilaria crassna* Pierre ex H.Lec. (kritsana) trees. Acta Hortic. 2005; 676: 161–66.

[3] Convention on International Trade in Endangered Species of Wild Fauna and Flora (CITES). Consideration of Proposals for Amendment of Appendices I and II- *Aquilaria* spp. and *Gyrinops* spp. Thirteenth meeting of the Conference of the Parties, Bangkok, Thailand, 2–14 October 2004.

[4] Chinese Pharmacopoeia Commission. Pharmacopeia of the People’s Republic of China. Volume 1 China Medical Science Press; 2010.

[5] Compton J, Ishihara A. The use and trade of agarwood in Japan. TRAFFIC Southeast Asia and TRAFFIC East Asia-Japan; 2004.

[6] Antonopoulou M, Compton J, Perry LS, Al-Mubarak R. The trade and use of agarwood (Oudh) in the United Arab Emirates. Selangor: TRAFFIC Southeast Asia; 2010.

[7] Mabberley DJ. Mabberley's plant-book: a portable dictionary of plants, their classifications, and uses. Cambridge University Press; 2008.

[8] The Plant List. Version 1.1. 2013. Available: http://www.theplantlist.org/. Accessed 5 October 2015.

[9] Barden A, Awang Anak N, Mulliken T, Song M. Heart of the matter: agarwood use and trade and CITES implementation for *Aquilaria malaccensis*. Cambridge: Traffic International; 2000.

[10] Soehartono T, Newton AC. Reproductive ecology of *Aquilaria spp*. in Indonesia. Forest Ecol Manag. 2001; 152: 59–71.

[11] Nakashima EM, Nguyen MT, Tran QL, Kadota S. Field survey of agarwood cultivation at Phu Quoc Island in Vietnam. Journal of Traditional Medicines. 2005; 22: 296–300.

[12] Yang DJ, Qiu Q, Wen J. Preliminary study on *Aquilaria sinensis* cultivate in mountainous area and the growth rule of young plantation. Guangxi Forestry Science. 2007; 4: 008.

[13] Lok EH, Yahya AZ. The growth performance of plantation grown *Aquilaria malaccensis* in Peninsular Malaysia. Journal of Tropical Forest Science. 1996; 8: 573–575.

[14] Lim TW, Awang Anak N. Wood for the Trees: A review of the agarwood (gaharu) trade in Malaysia Traffic Southeast Asia; 2010.

[15] Gasson P. How precise can wood identification be? Wood anatomy’s role in support of the legal timber trade, especially CITES. IAWA J. 2011; 32: 137–154.

[16] Jiao L, Yin Y, Cheng Y, Jiang X. DNA barcoding for identification of the endangered species *Aquilaria sinensis*: comparison of data from heated or aged wood samples. Holzforschung. 2014; 68: 487–494.

[17] Eurlings MCM, Gravendeel B. *Trn*L-*trn*F sequence data imply paraphyly of *Aquilaria* and *Gyrinops* (Thymelaeaceae) and provide new perspectives for agarwood identification. Plant Syst Evol. 2005; 254: 1–12.

[18] Ito M, Honda G. Taxonomical identification of agarwood-producing species. Natural Medicines. 2005; 59: 104–112.

[19] Lee SY, Weber JS, Mohamed R. Genetic variation and molecular authentication of selected *Aquilaria* species from natural populations in Malaysia using RAPD and SCAR marker. Asian J Plant Sci. 2011; 10: 202–211.

[20] Hebert PD, Cywinska A, Ball SL. Biological identifications through DNA barcodes. Proc Biol Sci. 2003; 270: 313–321. 12614582 10.1098/rspb.2002.2218 PMC1691236

[21] Che J, Chen HM, Yang JX, Jin JQ, Jiang KE, Yuan ZY, et al Universal COI primers for DNA barcoding amphibians. Mol Ecol Resour. 2012; 12: 247–258. 10.1111/j.1755-0998.2011.03090.x 22145866

[22] Hebert PD, Stoeckle MY, Zemlak TS, Francis CM. Identification of birds through DNA barcodes. PLOS Biol. 2004; 2: 1657–1663. 10.1371/journal.pbio.0020312 PMC518999 15455034

[23] Ward RD, Hanner R, Hebert PD. (2009). The campaign to DNA barcode all fishes, FISH-BOL. J Fish Biol. 2009; 74: 329–356. 10.1111/j.1095-8649.2008.02080.x 20735564

[24] Ball SL, Armstrong KF. DNA barcodes for insect pest identification: a test case with tussock moths (Lepidoptera: Lymantriidae). Can J For Res. 2006; 36: 337–350.

[25] Borisenko AV, Lim BK, Ivanova NV, Hanner RH, Hebert PD. DNA barcoding in surveys of small mammal communities: a field study in Suriname. Mol Ecol Resour. 2008; 8: 471–479. 10.1111/j.1471-8286.2007.01998.x 21585824

[26] Hollingsworth PM, Forrest LL, Spouge JL, Hajibabaei M, Ratnasingham S, van der Bank M, et al A DNA barcode for land plants. Proc Natl Acad Sci U S A. 2009; 106: 12794–12797. 10.1073/pnas.0905845106 19666622 PMC2722355

[27] Kress WJ, Erickson DL. A two-locus global DNA barcode for land plants: the coding *rbcL* gene complements the non-coding *trnH-psbA* spacer region. PLOS One. 2007; 2: e508 17551588 10.1371/journal.pone.0000508 PMC1876818

[28] Li DZ, Gao LM, Li HT, Wang H, Ge XJ, Liu JQ, et al Comparative analysis of a large dataset indicates that internal transcribed spacer (ITS) should be incorporated into the core barcode for seed plants. Proc Natl Acad Sci U S A. 2011; 108: 19641–19646. 10.1073/pnas.1104551108 22100737 PMC3241788

[29] Yao H, Song J, Liu C, Luo K, Han J, Li Y, et al Use of ITS2 region as the universal DNA barcode for plants and animals. PLOS One. 2010; 5: e13102 10.1371/journal.pone.0013102 20957043 PMC2948509

[30] Chen S, Pang X, Song J, Shi L, Yao H, Han J, Leon C. A renaissance in herbal medicine identification: From morphology to DNA. Biotechnol Adv. 2014; 32: 1237–1244. 10.1016/j.biotechadv.2014.07.004 25087935

[31] Song J, Shi L, Li D, Sun Y, Niu Y, Chen Z, et al Extensive pyrosequencing reveals frequent intra-genomic variations of internal transcribed spacer regions of nuclear ribosomal DNA. PLOS One. 2012; 7: e43971 10.1371/journal.pone.0043971 22952830 PMC3431384

[32] Xin T, Yao H, Gao H, Zhou X, Ma X, Xu C, et al Super food *Lycium barbarum* (Solanaceae) traceability via an internal transcribed spacer 2 barcode. Food Res Int. 2013; 54: 1699–1704.

[33] Techen N, Parveen I, Pan Z, Khan IA. DNA barcoding of medicinal plant material for identification. Curr Opin Biotechnol. 2014; 25: 103–110. 10.1016/j.copbio.2013.09.010 24484887

[34] Li X, Yang Y, Henry RJ, Rossetto M, Wang Y, Chen S. Plant DNA barcoding: from gene to genome. Biological Reviews. 2015; 90: 157–166. 10.1111/brv.12104 24666563

[35] Xin T, Li X, Yao H, Lin Y, Ma X, Cheng R, et al 2015 Survey of commercial *Rhodiola* products revealed species diversity and potential safety issues. Sci Rep. 2015; 5: 8337 10.1038/srep08337 25661009 PMC4321177

[36] Van der Bank M, Fay MF, Chase MW. Molecular phylogenetics of Thymelaeaceae with particular reference to African and Australian genera. Taxon. 2002; 51: 329–339.

[37] Rautenbach M. *Gnidia* L. (Thymelaeaceae) is not monophyletic: taxonomic implications for *Gnidia* and its relatives in Thymelaeoideae Doctoral dissertation, The University of Johannesburg 2008 Available: https://ujdigispace.uj.ac.za/handle/10210/784

[38] Nithaniyal S, Newmaster SG, Ragupathy S, Krishnamoorthy D, Vassou SL, Parani M. DNA barcode authentication of wood samples of threatened and commercial timber trees within the tropical dry evergreen forest of India. PLOS One. 2014; 9: e107669 10.1371/journal.pone.0107669 25259794 PMC4178033

[39] Sass C, Little DP, Stevenson DW, Specht DC. DNA barcoding in the cycadales: testing the potential of proposed barcoding markers for species identification of cycads. PLOS One. 2007; 2: e115. 10.1371/journal.pone.0001154 PMC2063462 17987130

[40] Sang T, Crawford DJ, Stuessy TF. Chloroplast DNA phylogeny, reticulate evolution and biogeography of *Paeonia* (Paeoniaceae). Am. J. Bot. 1997; 4: 1120–1136. 21708667

[41] Tate JA, Simpton BB. Paraphyly of *Tarasa* (Malvaceae) and diverse origins of the polyploidy species. Systematic Botany. 2003; 28: 723–737.

[42] Taberlet P, Gielly L, Pautou G, Bouvet J. Universal primers for amplification of three non-coding regions of chloroplast DNA. Plant Mol Biol. 1991; 17: 1105–1109. 1932684 10.1007/BF00037152

[43] Baldwin BG. Phylogenetic utility of the internal transcribed spacers of nuclear ribosomal DNA in plants: an example from the Compositae. Mol Phylogenet Evol. 1992; 1: 3–16. 1342921 10.1016/1055-7903(92)90030-k

[44] Chen S, Yao H, Han J, Liu C, Song J, Shi L, et al Validation of the ITS2 region as a novel DNA barcode for identifying medicinal plant species. PLOS One. 2010; 5: e8613 10.1371/journal.pone.0008613 20062805 PMC2799520

[45] Kimura M. A simple method for estimating evolutionary rates of base substitutions through comparative studies of nucleotide sequences. J Mol Evol. 1980; 16: 111–120. 7463489 10.1007/BF01731581

[46] Tamura K, Stecher G, Peterson D, Filipski A, Kumar S. MEGA6: Molecular Evolutionary Genetics Analysis Version 6.0. Mol Biol Evol. 2013; 30: 2725–2729. 10.1093/molbev/mst197 24132122 PMC3840312

[47] Meier R, Shiyang K, Vaidya G, Ng PK. DNA barcoding and taxonomy in Diptera: a tale of high intraspecific variability and low identification success. Syst Biol. 2006; 55: 715–728. 17060194 10.1080/10635150600969864

[48] Herber BE. Pollen morphology of the Thymelaeaceae in relation to its taxonomy. Plant Syst Evol, 2002; 232: 107–121.

[49] Herber BE. Thymelaeaceae In Kubitzki K, Bayer C, editors. Flowering Plants: Dicotyledons. Springer Berlin Heidelberg; 2003 pp. 373–396.

[50] Kiet LC, Kessler PJ, Eurlings M. A new species of Aquilaria (Thymelaeaceae) from Vietnam. Blumea. 2005; 50: 135–141.

[51] Shen YJ, Tan XM, Zhao X, Pang QH, Zhao SJ. Ribosomal DNA ITS sequence analysis of *Aquilaria sinensis* from different geographical origin in China. China Journal of Traditional Chinese Medicine and Pharmacy. 2009; 4: 46.

[52] Niu XL, Ji KP, Lu GQ. Preliminary studies on identification of *Aquilaria sinensis* (L.) Gilg by the PCR product of rDNA ITS sequencing. Guangdong Nong Ye Ke Xue. 2010; 37: 167–9.

[53] Lee SY, Mohamed R. Rediscovery of *Aquilaria rostrata* (Thymelaeaceae), a species thought to be extinct, and notes on *Aquilaria* conservation in Peninsular Malaysia. Blumea. 2016; 61: 13–19.

[54] Ashfaq M, Asif M, Anjum ZI, Zafar Y. Evaluating the capacity of plant DNA barcodes to discriminate species of cotton (*Gossypium*: Malvaceae). Mol Ecol Resour. 2013; 13: 573–582 10.1111/1755-0998.12089 23480447

[55] Hou D. Notes on some Asiatic species of *Aquilaria* (Thymelaeaceae). Blumea. 1964; 12: 285–288.

[56] Mohamed R, Tan HY, Siah CH. A real-time PCR method for the detection of *trn*L-*trn*F sequence in agarwood and products from *Aquilaria* (Thymelaeaceae). Con Genet Resour. 2012; 4: 803–806.

[57] Valentini A, Pompanon F, Taberlet P. DNA barcoding for ecologists. Trends Ecol Evol. 2009; 24: 110–117. 10.1016/j.tree.2008.09.011 19100655

