# Supplementary material for: DNA Barcoding of the Endangered Aquilaria (Thymelaeaceae) and Its Application in Species Authentication of Agarwood Products Traded in the Market
Source: PLoS One. 2016 Apr 29;11(4):e0154631. doi: 10.1371/journal.pone.0154631 (PMC4851384; doi:10.1371/journal.pone.0154631)
Supplement: S1 Fig — (a) matK, (b) rbcL, (c) rpoB, (d) rpoC1, (e) psbA-trnH, (f) trnL-trnF, (g) ITS, (h) ITS2, (i) trnL-trnF+ITS, (j) trnL-trnF+ITS2, (k) trnL-trnF+psbA-trnH, (l) trnL-trnF+psbA-trnH+ITS, (m) trnL-trnF+psbA-trnH+ITS2, (n)matK+trnL-trnF, (o) matK+rbcL+trnL-trnF, (p) matK+ITS, (q) matK+ITS2, (r) matK+rbcL+ITS, (s) matK+rbcL+ITS2, (t) matK+trnL-trnF+ITS, (u) matK+trnL-trnF+ITS2, (v) matK+rbcL+trnL-trnF+ITS, (w) matK+rbcL+trnL-trnF+ITS2, (x) matK+rbcL and (y) rbcL+trnL-trnF+ITS (PDF) [file pone.0154631.s001.pdf]

S1 Fig. Distribution of intra- and inter-specific Kimura 2-parameter (K2P) distances among all samples for the eight candidate loci and their combinations. (a) *mat*K, (b) *rbc*L, (c) *rpo*B, (d) *rpo*C1, (e) *psb*A-*trn*H, (f) *trn*L-*trn*F, (g) ITS, (h) ITS2, (i) *trn*L-*trn*F+ITS, (j) *trn*L-*trn*F+ITS2, (k) *trn*L-*trn*F+*psb*A-*trn*H, (l) *trn*L-*trn*F+*psb*A-*trn*H+ITS, (m) *trn*L-*trn*F+*psb*A-*trn*H+ITS2, (n) *mat*K+*trn*L-*trn*F, (o) *mat*K+*rbc*L+*trn*L-*trn*F, (p) *mat*K+ITS, (q) *mat*K+ITS2, (r) *mat*K+*rbc*L+ITS, (s) *mat*K+*rbc*L+ITS2, (t) *mat*K+*trn*L-*trn*F+ITS, (u) *mat*K+*trn*L-*trn*F+ITS2, (v) *mat*K+*rbc*L+*trn*L-*trn*F+ITS, (w) *mat*K+*rbc*L+*trn*L-*tr*nF+ITS2, (x) *mat*K+*rbc*L and (y).*rbc*L+*trn*L-*trn*F+ITS

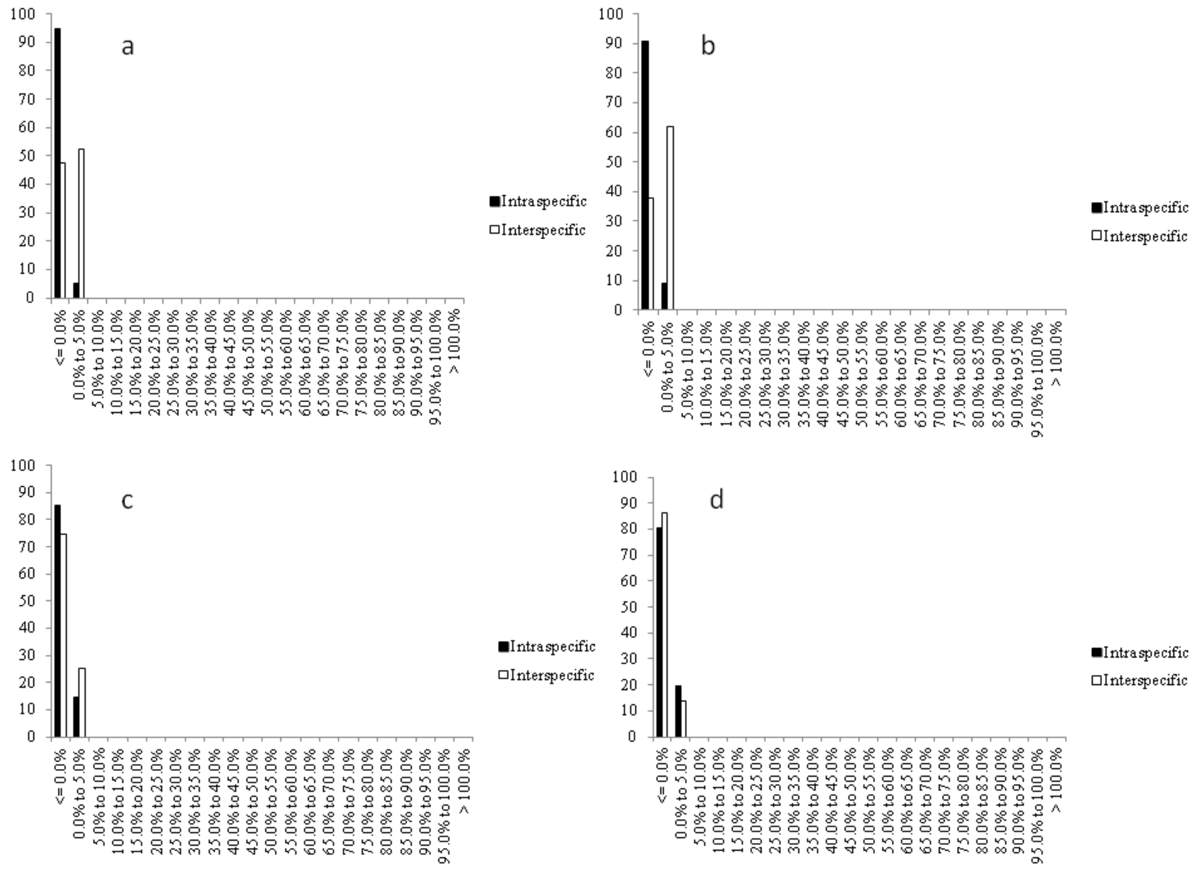

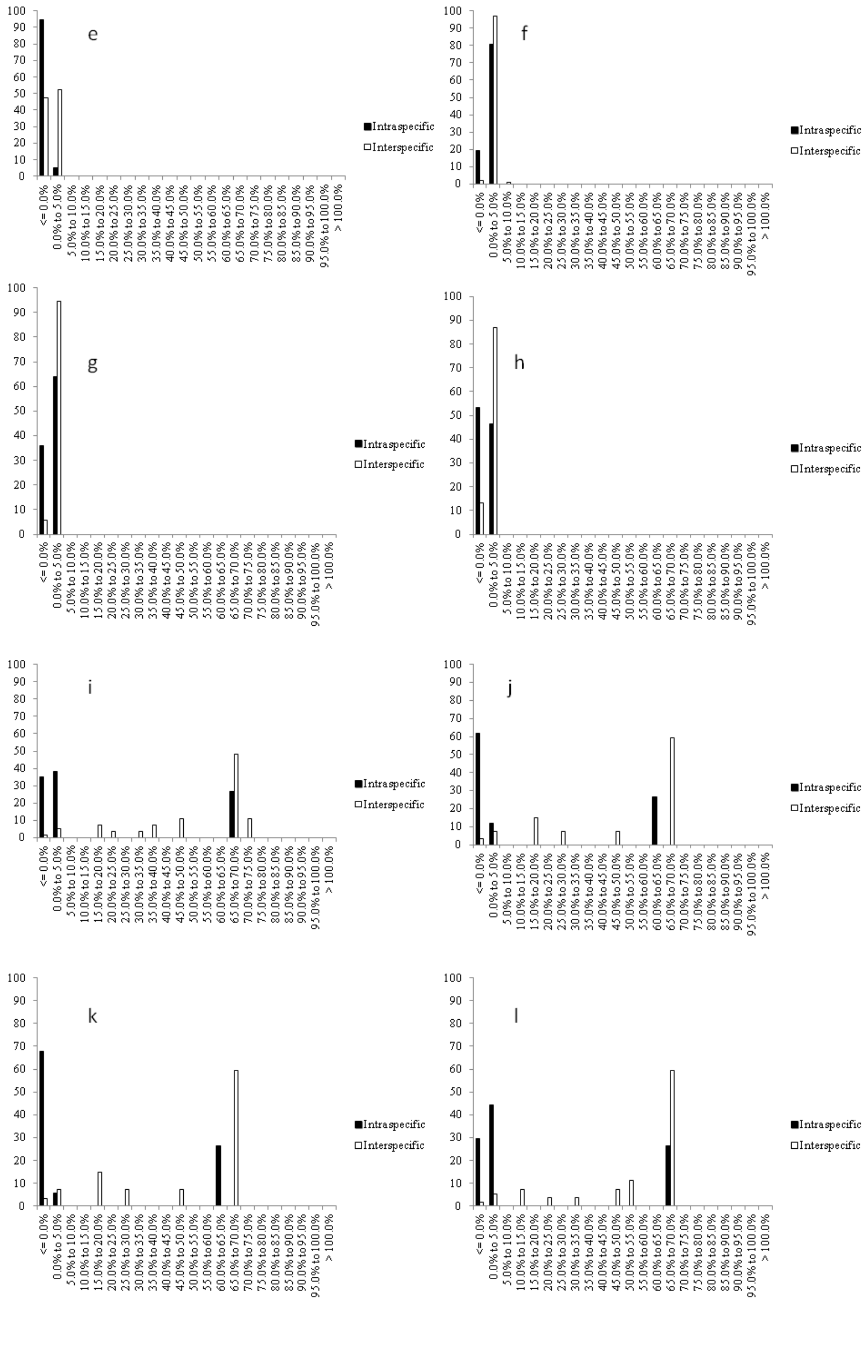
e
f
g
h
i
j
k
l
Intraspecific
Interspecific
0
10
20
30
40
50
60
70
80
90
100
<= 0.0%
0.0% to 5.0%
5.0% to 10.0%
10.0% to 15.0%
15.0% to 20.0%
20.0% to 25.0%
25.0% to 30.0%
30.0% to 35.0%
35.0% to 40.0%
40.0% to 45.0%
45.0% to 50.0%
50.0% to 55.0%
55.0% to 60.0%
60.0% to 65.0%
65.0% to 70.0%
70.0% to 75.0%
75.0% to 80.0%
80.0% to 85.0%
85.0% to 90.0%
90.0% to 95.0%
95.0% to 100.0%
> 100.0%

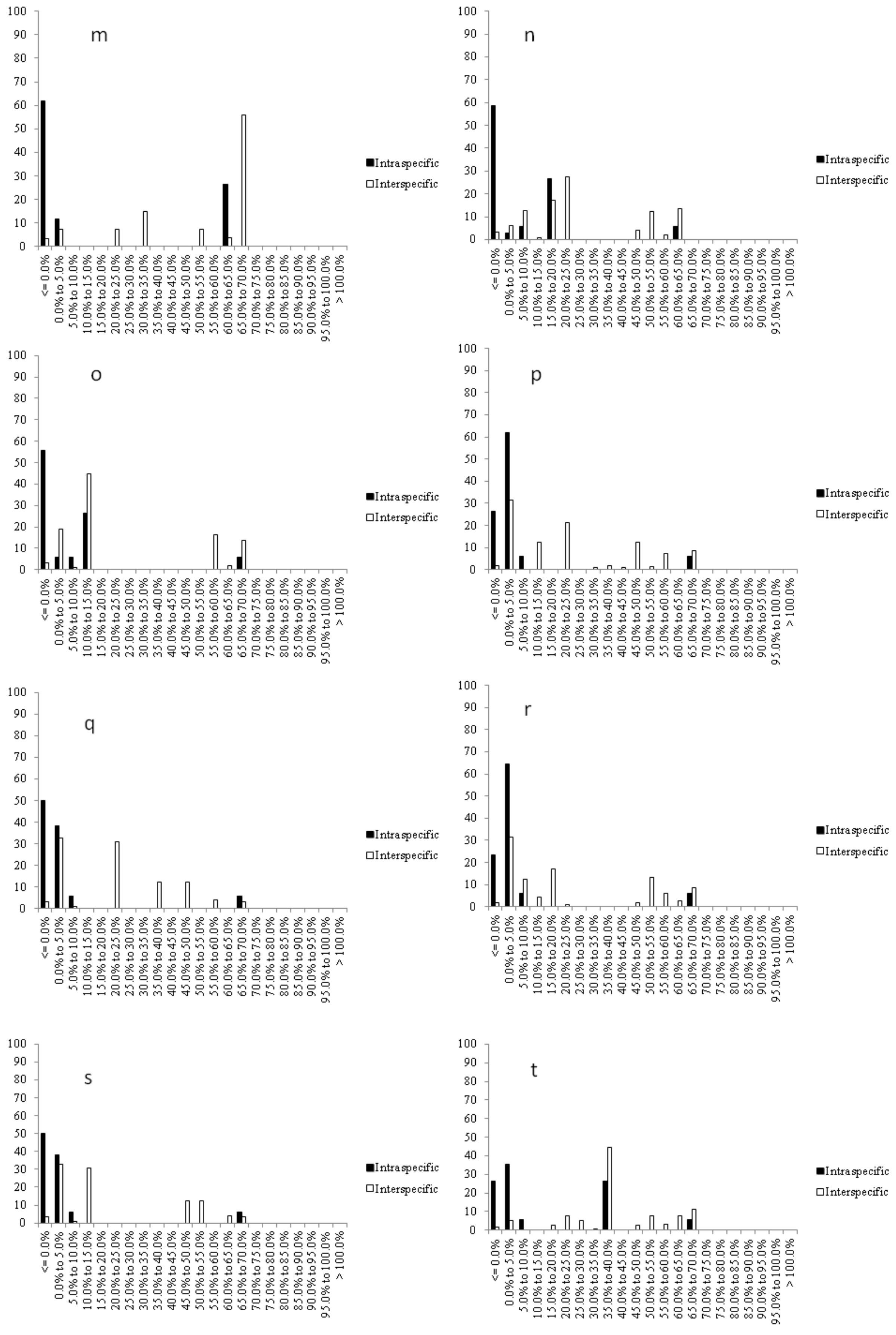
m
Intraspecific
Interspecific
n
Intraspecific
Interspecific
o
Intraspecific
Interspecific
p
Intraspecific
Interspecific
q
Intraspecific
Interspecific
r
Intraspecific
Interspecific
s
Intraspecific
Interspecific
t
Intraspecific
Interspecific
<= 0.0%
0.0% to 5.0%
5.0% to 10.0%
10.0% to 15.0%
15.0% to 20.0%
20.0% to 25.0%
25.0% to 30.0%
30.0% to 35.0%
35.0% to 40.0%
40.0% to 45.0%
45.0% to 50.0%
50.0% to 55.0%
55.0% to 60.0%
60.0% to 65.0%
65.0% to 70.0%
70.0% to 75.0%
75.0% to 80.0%
80.0% to 85.0%
85.0% to 90.0%
90.0% to 95.0%
95.0% to 100.0%
> 100.0%

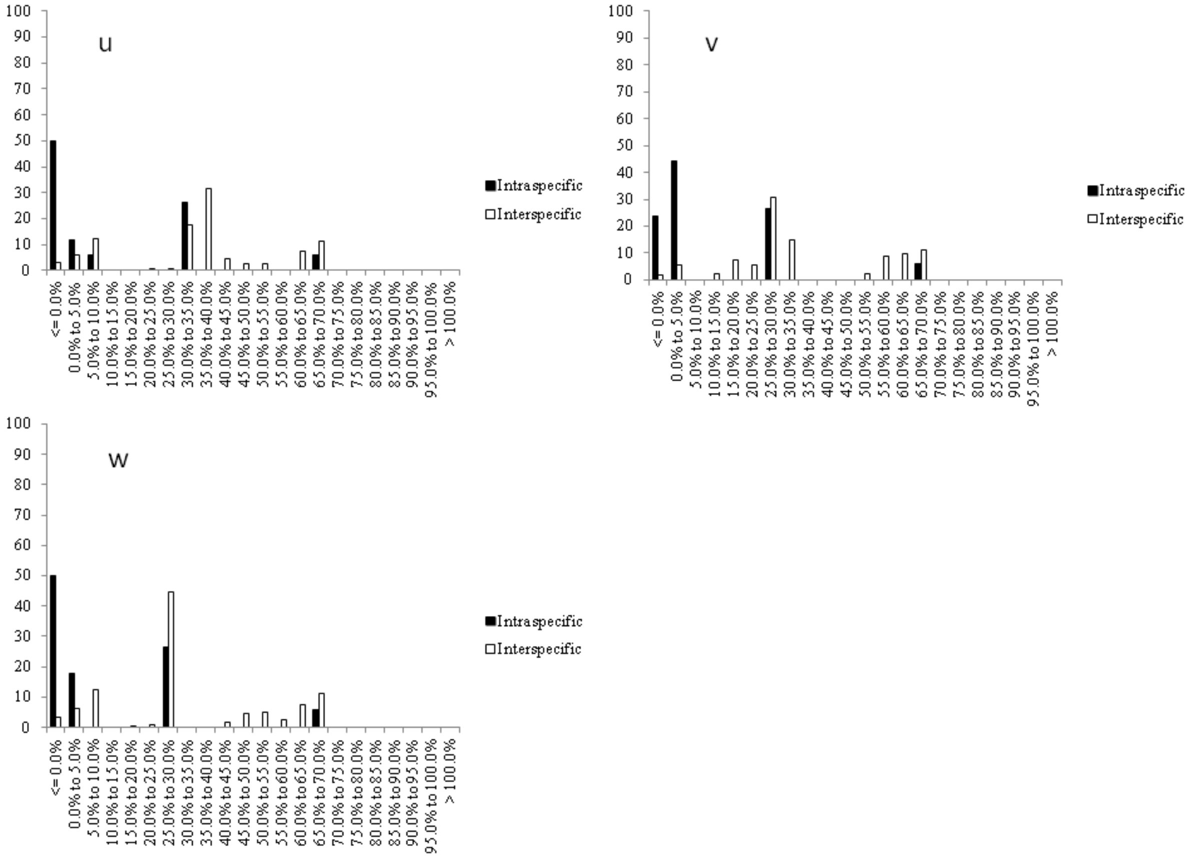
u
v
w
100
90
80
70
60
50
40
30
20
10
0
<= 0.0%
0.0% to 5.0%
5.0% to 10.0%
10.0% to 15.0%
15.0% to 20.0%
20.0% to 25.0%
25.0% to 30.0%
30.0% to 35.0%
35.0% to 40.0%
40.0% to 45.0%
45.0% to 50.0%
50.0% to 55.0%
55.0% to 60.0%
60.0% to 65.0%
65.0% to 70.0%
70.0% to 75.0%
75.0% to 80.0%
80.0% to 85.0%
85.0% to 90.0%
90.0% to 95.0%
95.0% to 100.0%
> 100.0%
Intraspecific
Interspecific
